# Ethnic and Gender Differentials in Non-Communicable Diseases and Self-Rated Health in Malaysia

**DOI:** 10.1371/journal.pone.0091328

**Published:** 2014-03-06

**Authors:** Jane K. L. Teh, Nai Peng Tey, Sor Tho Ng

**Affiliations:** Population Studies Unit, Faculty of Economics and Administration, University of Malaya, Kuala Lumpur, Malaysia; Endocrine Research Center (Firouzgar), Institute of Endocrinology and Metabolism, Iran (Republic of Islamic)

## Abstract

**Objectives:**

This paper examines the ethnic and gender differentials in high blood pressure (HBP), diabetes, coronary heart disease (CHD), arthritis and asthma among older people in Malaysia, and how these diseases along with other factors affect self-rated health. Differentials in the prevalence of non-communicable diseases among older people are examined in the context of socio-cultural perspectives in multi-ethnic Malaysia.

**Methods:**

Data for this paper are obtained from the 2004 Malaysian Population and Family Survey. The survey covered a nationally representative sample of 3,406 persons aged 50 and over, comprising three main ethnic groups (Malays, Chinese and Indians) and all other indigenous groups. Bivariate analyses and hierarchical logistic regression were used in the analyses.

**Results:**

Arthritis was the most common non-communicable disease (NCD), followed by HBP, diabetes, asthma and CHD. Older females were more likely than males to have arthritis and HBP, but males were more likely to have asthma. Diabetes and CHD were most prevalent among Indians, while arthritis and HBP were most prevalent among the Indigenous groups. Older people were more likely to report poor health if they suffered from NCD, especially CHD. Controlling for socio-economic, health and lifestyle factors, Chinese were least likely to report poor health, whereas Indians and Indigenous people were more likely to do so. Chinese that had HBP were more likely to report poor health compared to other ethnic groups with the same disease. Among those with arthritis, Indians were more likely to report poor health.

**Conclusion:**

Perceived health status and prevalence of arthritis, HBP, diabetes, asthma and CHD varied widely across ethnic groups. Promotion of healthy lifestyle, early detection and timely intervention of NCDs affecting different ethnic groups and gender with socio-cultural orientations would go a long way in alleviating the debilitating effects of the common NCDs among older people.

## Introduction

One of the major challenges of population aging is meeting the healthcare needs of rapidly increasing number of older persons suffering from various health problems. Non-communicable diseases (NCDs) are the most common health problems, and are the primary causes of death in many countries. Cardiovascular diseases, cancers, chronic respiratory diseases and diabetes are the top four NCDs which have resulted in highest number of deaths, especially in low and middle-income countries [1]. NCD patients suffer health and physical limitations and are also burdened by the costly treatment of these diseases [1], [2]. Complications from NCDs such as diabetes are costly to treat, and are rarely curable [3].

Unhealthy lifestyles and diet such as tobacco use, lack of physical activity, poor diet and excessive use of alcohol increase the risk of NCDs [1], [2]. Certain diseases tend to be more prevalent among some ethnic groups. For instance, high blood pressure is more common among Blacks in a Western population, and diabetes and cardiovascular diseases are more prevalent among Indians [4], [5].

The single-item indicator of self-rated health is often used to indicate the overall health status of individuals [6], [7], [8]. Despite its simplicity, it is inherently a multidimensional concept capturing dimensions of physical, functional, coping and well-being [9], [10]. Several studies have focused on factors affecting health assessments. Chronic diseases, chronic pain and physical disability are significant predictors of self-rated health [11], [12], [13], [14]. Perceived health also varies according to age, gender, ethnicity, education, income, unemployment, and lifestyle factors such as smoking and physical inactivity [15], [16], [17], [18], [19], [20]. The ethnic differentials in health assessments have rarely been addressed, particularly in low and middle-income countries. Several studies found that poor self-rated health is closely associated with mortality, especially among the elderly [21], [22], [23], [24]. Hence, there has been growing interest in the study of self-rated health as an indicator for the health status of a population.

Multi-ethnic Malaysia provides an excellent setting for a study on the differentials of health problems of older people, from a sociocultural perspective. Malaysia is a South-east Asian, middle-income nation. The western parts (Peninsular Malaysia) and the eastern parts (Sabah and Sarawak) are separated by South China Sea. Of a total population of 28.3 million in 2010, the Malays made up 55%, Chinese 24%, Indians 7%, other Indigenous people 13% and others 1%. The Malays, Chinese and Indians are the predominant ethnic groups in Peninsular Malaysia, whereas Indigenous groups are mostly from the states of Sabah and Sarawak in East Malaysia [25]. Ethnic groups in Malaysia have diverse cultures, religions and background characteristics [26]. Islam is the national religion. All Malays are Muslim, 75.9% of the Chinese are Buddhist and 9.6% are Christian, 84.5% of the Indians are Hindu and 7.7% are Christian, and about half of the Indigenous people are Christian and 36.3% are Muslim [27]. Thus, a comparison among these ethnic groups may reveal socio-cultural differentials in illness patterns.

The objectives of this study are to examine 1) the ethnic and gender differentials in the prevalence of selected NCDs, and 2) the impact of NCDs and other factors affecting poor self-rated health, among older persons within a multi-ethnic setting. A better understanding of the ethnic and gender differentials in NCDs and poor self-rated health is needed to target high risk groups in the provision of services, and to arrest the growing burden of NCDs. This study also seeks to contribute to the literature on the health differentials across the 3 largest ethnic groups in the world, i.e., the Malays, Chinese and Indians.

## Methods

### Ethics Statement

This study utilized survey data collected by the National Population and Family Development Board (NPFDB) of Malaysia (http://www.lppkn.gov.my/index.php?lang=en). With the approval of the director general of NPFDB, we signed a Terms of Reference Contract and were given permission by the Research Committee of the NPFDB to use the data for research and publication. The survey was conducted according to the existing laws and regulations, and data has already been de-identified by NPFDB. Therefore we were exempted from obtaining ethical approval.

### Data Sources

Data for this study are taken from the 2004 Malaysian Population and Family Survey (MPFS-4). The MPFS-4 was conducted in 2004 by the NPFDB, under the purview of the Ministry of Women, Family and Community Development of Malaysia, with funding from the Economic Planning Unit and technical assistance in sample selection from the Department of Statistics of Malaysia. The MPFS-4 sample was selected using a stratified multistage sampling design, and the survey was fielded between July 2004 and September 2004. The Kish method was used in the selection of senior citizens within the selected households. Participants gave oral consent and face-to-face interviews were conducted by trained personnel. The MPFS was conducted decennially since 1974. The 2004 MPFS was the first covering Peninsular Malaysia, Sabah and Sarawak [28], [29], [30]. This study is confined to Malaysian citizens aged 50 and above.

### Measures

#### Self-rated health

The 3-category self-rated health variable (*1 = Good, 2 = Average, 3 = Poor*) was recoded into a binary variable to indicate poor health (*0 = Good or average health assessment, 1 = Poor health assessment*).

#### NCDs

Respondents were asked if they were suffering from 5 types of diseases: 1) high blood pressure (HBP), 2) diabetes, 3) coronary heart disease (CHD), 4) arthritis, and 5) asthma. Responses for each of these diseases were coded as binary variables (*0 = No, 1 = Yes*).

#### Physical limitations

Respondents were asked if they were unable to perform a list of tasks: 1) feed oneself, 2) bath unassisted, 3) get dressed unassisted, 4) go to the toilet unassisted, 5) exercise, 6) do housework, 7) attend religious gatherings, and 8) grocery shopping. The number of physical limitations were totaled and recoded into a 3-category variable (*1 = none, 2 = “1 or 2 limitations”, 3 = “3 or more limitations”*).

#### Demographic variables

Demographic variables include respondent’s age (1 = 50–59 years, 2 = 60–69 years, 3 = 70+ years), gender (0 = Female, 1 = Male), ethnicity (1 = Malay, 2 = Chinese, 3 = Indian, 4 = Indigenous), marital status (0 = Unmarried, 1 = Married) and place of residence (0 = Rural, 1 = Urban). Respondents who were widowed, divorced, separated, or never married were categorized as unmarried.

#### Socio-economic status (SES)

Variables reflecting respondent’s SES include education (*1 = None, 2 = Primary, 3 = Secondary and above*), current work/employment status (*0 = Not working, 1 = Working*), and number of other income sources (*1 = 0 source, 2 = 1 or 2 sources, 3 = 3 or more sources*). In the survey, respondents were asked if they had any inheritance (house, company, land, etc.), savings in the Employees Provident Fund (a defined-contribution public pension plan), pension, rewards/remunerations, savings in bank, savings in ‘tabung haji’(the Malaysian hajj pilgrims fund board), share investments and insurance. These sources of income were totaled and recoded into a 3-category variable.

#### Lifestyle factors

Variables indicating lifestyle factors include exercise (1 = never, 2 = 1 to 3 times a week, 3 = 4 or more times a week), and smoking/tobacco use (1 = Non-smoker, 2 = Previous smoker, 3 = Current smoker).

### Statistical Analysis

We began by examining the ethnic differentials in demographic, socio-economic, lifestyle and health characteristics, with tests of association between ethnicity and these variables. This was followed by an examination of the prevalence of the 5 diseases (HBP, diabetes, CHD, arthritis and asthma) according to ethnicity, gender and age of respondents. As the independent variables are inter-related, separate binary logistic regressions [31] were carried out to examine net effects of ethnicity, gender, age and socio-economic variables on each NCD at *p*<0.05. Finally, hierarchical binary logistic regression [31] was run to examine the impact of NCDs, and demographic, socio-economic and lifestyle factors, affecting self-rated health. Age, gender, ethnicity, marital status and place of residence were entered into the model in the first step, socio-economic variables in the second step, physical limitations and NCDs in the third step and lifestyle factors in the fourth step. Two-way interaction terms between ethnicity and each disease were tested and assessed through likelihood ratio tests at *p*<0.05. The final model with significant interaction terms were confirmed through Wald statistics. In multivariate analyses, we weighted the dataset to reflect the population distribution of Malays, Chinese, Indians and Indigenous groups [25]. Data were analyzed using SPSS for Windows version 19.

Odds ratio greater than 1 is interpreted as how many times an event was more likely to occur, compared to the reference group. For odds ratio of less than 1, its inverse is interpreted as how many times an event is less likely to occur as compared to the reference group.

## Results

The sample of 3,406 respondents consisted of 46% Malays, 25% Chinese, 5% Indians and 24% Indigenous people (Table 1). The majority of respondents were in their 50 s-60 s (80.6%). Females made up 66.2% of the sample, and 61.6% were currently married. A little more than half (52.3%) were living in rural areas. Malays and Indigenous people were concentrated in rural areas whereas the Chinese and Indians were mainly from the urban areas. The majority had primary or no schooling (79.8%), and the proportion of respondents with no formal schooling was much higher among the Indigenous people. About one third of the Chinese and Indians had at least secondary education. About two thirds (63.9%) were not working and this percentage was significantly lower among the Indigenous people. Slightly more than three quarters (77.1%) had at least 1 source of income, with the Malays relatively more likely to have multiple sources of income. In terms of lifestyle habits, 61.3% did not exercise and 67.9% were non-smokers. Chinese and Indians were relatively more likely than Malays and Indigenous people to exercise more than 3 times a week, whereas the prevalence of smoking was highest among the Indigenous people. Health-wise, 82.7% reported good or average health. The Malays were more likely than the rest to have multiple physical limitations. The Chinese were more likely to report good health, whereas the Indians and Indigenous people were more likely to perceive themselves to be in poor health. Except for age, significant associations were found between ethnicity and all other variables.

**Table 1:** Characteristics of respondents according to ethnic groups.

	Ethnic Groups					
Attributes	Malays *n* = 1554	Chinese *n* = 854	Indians *n* = 173	Indigenous *n* = 825	Total n = 3406	Pearson Chi-square *p* value
**Age**						
**50** **s**	46.0	46.6	53.8	42.4	45.7	
**60** **s**	35.3	33.7	31.2	36.1	34.9	
**70** **s +**	18.7	19.7	15.0	21.5	19.4	0.125
**Gender**						
**Female**	68.7	63.2	73.4	63.0	66.2	
**Male**	31.3	36.8	26.6	37.0	33.8	0.001
**Marital status**						
**Unmarried**	39.3	34.3	44.5	39.9	38.4	
**Married**	60.7	65.7	55.5	60.1	61.6	0.019
**Residence**						
**Rural**	59.1	21.7	20.8	77.9	52.3	
**Urban**	40.9	78.3	79.2	22.1	47.7	<0.001
**Education**						
**No schooling**	34.3	23.6	20.9	70.9	39.8	
**Primary**	47.3	44.3	43.6	21.1	40.0	
**Secondary +**	18.4	32.1	35.5	8.0	20.2	<0.001
**Work status**						
**Not working**	67.0	62.4	75.1	57.1	63.9	
**Still working**	33.0	37.6	24.9	42.9	36.1	<0.001
**Other sources of income**						
**0 source**	21.0	18.4	25.4	30.4	22.9	
**1–2 sources**	46.8	59.0	50.3	50.5	50.9	
**3+ sources**	32.2	22.6	24.3	19.0	26.2	<0.001
**Exercise**						
**0 times**	59.6	46.3	43.5	83.8	61.3	
**1–3timesa week**	21.4	23.1	25.9	12.3	19.8	
**4+ times a week**	19.0	30.6	30.6	3.9	18.8	<0.001
**Smoker**						
**Non smoker**	69.3	73.8	83.2	55.8	67.9	
**Previous smoker**	12.2	12.2	8.1	17.8	13.4	
**Current smoker**	18.5	14.1	8.7	26.4	18.8	<0.001
**Physical limitations**						
**0 limits.**	75.5	85.0	82.1	80.8	79.5	
**1–2 limits.**	14.4	9.5	9.8	12.7	12.5	
**3+ limits.**	10.2	5.5	8.1	6.4	8.0	<0.001
**Self-rated health**						
**Good**	39.5	54.2	36.4	33.3	41.5	
**Average**	45.0	34.4	38.7	41.6	41.2	
**Poor**	15.4	11.4	24.9	25.1	17.2	<0.001

The overall prevalence of the 5 NCDs reported by respondents in this study was 35.8% for HBP, 14.1% for diabetes, 8% for CHD, 45.3% for arthritis and 13.2% for asthma (Table 2). Indigenous people were more likely than others to suffer from HBP, arthritis and asthma, while the Indians had the highest prevalence in diabetes and CHD. The prevalence of HBP, CHD, arthritis and asthma increased monotonically with age. However, the prevalence of diabetes was highest for those aged 60–69 among the females, Malays, Indians and Indigenous groups (data not shown). Other anomalies related to age and disease were: 1) CHD – higher prevalence among those aged 60–69 than those aged 70+ among Indians, 2) arthritis – highest prevalence among those aged 60–69 for the Malays, and decreased prevalence with advancing age among the Indians; and 3) asthma –decreasing prevalence with advance in age among the Indians (data not shown). Males were more likely than females to have diabetes, CHD and asthma, while the reverse was true for HBP and arthritis. With the exception of the Chinese, females were more likely than males to have HBP.

**Table 2:** Prevalence of 5 NCDs according to ethnicity, gender and age (percentage).

		HBP	Diabetes	CHD	Arthritis	Asthma
**Overall**		35.8	14.1	8.0	45.3	13.2
**Age Group**	50 s	30.8	12.9	5.4	39.7	10.1
	60 s	39.9	15.9	9.1	49.1	14.7
	70 s +	40.3	13.8	12.2	51.6	17.6
**Gender**	Female	37.7	14.0	7.9	50.5	11.5
	Male	32.2	14.4	8.4	35.2	16.3
**Malay**		34.3	15.5	7.1	46.7	13.7
	Female	38.1	15.2	6.7	51.4	11.7
	Male	25.7	16.4	8.0	36.7	18.1
**Chinese**		34.1	14.4	7.8	34.2	6.9
	Female	31.2	13.9	7.1	41.4	5.8
	Male	39.2	15.2	8.9	21.6	8.8
**Indian**		33.7	35.4	22.1	41.1	10.0
	Female	35.5	33.3	22.2	46.3	9.6
	Male	28.9	40.9	21.7	26.7	11.1
**Indigenous**		40.8	7.2	7.1	54.8	19.3
	Female	43.9	7.3	7.4	59.0	17.8
	Male	35.5	6.9	6.6	47.5	22.0

The odds of having each of the diseases are influenced by a multitude of factors (Table 3). As expected, the health problems tended to increase with advance in age. Respondents aged 70 and over were more likely than those aged 50–59 to suffer from HBP, CHD and arthritis, by as much as 1.3 times, 2.3 times and 1.7 times respectively. Males were less likely than females to suffer from HBP and arthritis, but more likely to suffer from asthma. There were also marked ethnic differentials in all 5 diseases. Indigenous people were 1.5 times more likely than the Malays to have HBP and arthritis, but they were 2.1 times less likely than the Malays to have diabetes. Compared to the Malays, Chinese were 1.6 times and 2 times less likely to have arthritis and asthma respectively, but Indians were 2.7 times and almost 4 times more likely to have diabetes and CHD respectively. Currently married persons had significantly lower odds of having asthma as compared to the unmarried (OR = 0.72). The effect of urban place of residence was only significant in predicting the odds of diabetes (OR = 1.32). The effect of higher education was significant in reducing the odds of suffering from arthritis (OR = 0.76) and asthma (OR = 0.62). Currently working significantly reduced the odds of suffering from all 5 diseases. Those with multiple sources of income had higher odds of HBP (OR = 1.38) and CHD (OR = 1.51), as compared to those with no sources of income.

**Table 3:** Logistic regression on selected NCDs (Odds ratios).

Variables	HBP	Diabetes	CHD	Arthritis	Asthma
**Age group (** ***ref:50*** ***s*** **)**					
**60** **s**	**1.40** (1.17, 1.67)	1.27 (1.00, 1.61)	**1.89** (1.37, 2.60)	**1.56** (1.31, 1.86)	1.16 (0.90, 1.50)
**70** **s +**	**1.32** (1.05, 1.67)	1.03 (0.75, 1.42)	**2.31** (1.56, 3.43)	**1.69** (1.34, 2.13)	1.15 (0.83, 1.58)
**Male**	**0.80** (0.67, 0.97)	1.16 (0.90, 1.48)	1.04 (0.76, 1.42)	**0.46** (0.38, 0.55)	**2.03** (1.57, 2.62)
**Ethnic (** ***ref: Malay*** **)**					
**Chinese**	1.00 (0.83, 1.22)	0.86 (0.66, 1.11)	1.11 (0.79, 1.56)	**0.62** (0.52, 0.75)	**0.51** (0.37, 0.70)
**Indian**	0.94 (0.70, 1.26)	**2.71** (1.98, 3.72)	**3.89** (2.66, 5.70)	0.81 (0.60, 1.08)	0.80 (0.51, 1.25)
**Indigenous**	**1.51** (1.20, 1.90)	**0.47** (0.31, 0.70)	1.04 (0.67, 1.59)	**1.52** (1.21, 1.91)	1.32 (0.98, 1.76)
**Married**	0.94 (0.79, 1.10)	1.07 (0.85, 1.34)	1.06 (0.79, 1.41)	1.16 (0.99, 1.37)	**0.72** (0.57, 0.92)
**Urban**	1.12 (0.95, 1.33)	**1.32** (1.05, 1.65)	1.09 (0.81, 1.45)	1.01 (0.86, 1.19)	0.89 (0.70, 1.14)
**Education (** ***ref: No schooling*** **)**					
**Primary**	1.04 (0.86, 1.25)	0.97 (0.75, 1.25)	0.97 (0.71, 1.33)	1.19 (0.99, 1.42)	0.79 (0.61, 1.02)
**Secondary +**	0.97 (0.76, 1.25)	1.00 (0.72, 1.39)	0.85 (0.55, 1.31)	**0.76** (0.59, 0.98)	**0.62** (0.42, 0.91)
**Work**	**0.57** (0.48, 0.67)	**0.57** (0.45, 0.73)	**0.69** (0.50, 0.94)	**0.81** (0.69, 0.95)	**0.68** (0.53, 0.88)
**Income sources (** ***ref: 0*** **)**					
**1–2**	**1.22** (1.01, 1.48)	0.94 (0.73, 1.22)	1.29 (0.92, 1.80)	1.16 (0.97, 1.39)	0.86 (0.66, 1.11)
**3+**	**1.38** (1.09, 1.73)	1.21 (0.89, 1.64)	**1.51** (1.01, 2.26)	1.22 (0.98, 1.53)	0.93 (0.68, 1.28)

Note: Figures in brackets show 95 percent confidence intervals. Odds ratio in bold indicate significance at 0.05.

Demographic, socio-economic factors, physical limitations, NCDs and lifestyle factors were found to be significant predictors of self-rated health (Table 4). With just demographic variables in the model, age, ethnicity and place of residence significantly affected self-rated health. Respondents aged 70 and over were 3 times more likely than those aged 50–59 to report poor health. The Indians and Indigenous people were more likely than the Malays to report poor health, by as much as 2.2 times and 1.7 times respectively. Urban residents were 1.3 times less likely than their rural counterparts to report poor health. In the second model, all 3 SES variables were significant in predicting the odds of perceived poor health, controlling for demographic variables. Higher education and more sources of income reduced the odds of poor self-rated health. Respondents who were working were 1.9 times less likely than the non-working people to report poor health. The effect of age and ethnicity remained significant, but urban-rural differentials in self-rated health disappeared after taking into account other variables in the model. The gender differential in self-rated health became significant after controlling for SES variables - the males were 1.4 times more likely than the females to perceive themselves to be in poor health.

**Table 4:** Hierarchical logistic regression on poor self-rated health (Odds ratio).

Variables	Model 1	Model 2	Model 3	Model 4	Model 5
**Age group (** ***ref: 50*** ***s*** **)**					
**60** **s**	**1.72** (1.35, 2.20)	**1.31** (1.01, 1.70)	1.01 (0.77, 1.33)	1.01 (0.77, 1.33)	1.05 (0.80, 1.39)
**70** **s +**	**3.02** (2.29, 3.97)	**1.86** (1.37, 2.52)	1.21 (0.85, 1.72)	1.21 (0.85, 1.73)	1.24 (0.87, 1.77)
**Male**	0.96 (0.76, 1.20)	**1.43** (1.11, 1.85)	**1.64** (1.24, 2.16)	**1.48** (1.08, 2.05)	**1.47** (1.06, 2.03)
**Ethnic (** ***ref: Malay*** **)**					
**Chinese**	0.79 (0.59, 1.04)	0.82 (0.61, 1.09)	0.93 (0.68, 1.26)	0.99 (0.73, 1.35)	**0.59** (0.35, 0.99)
**Indian**	**2.22** (1.57, 3.16)	**2.29** (1.60, 3.29)	**2.00** (1.34, 2.96)	**2.12** (1.42, 3.16)	1.11 (0.56, 2.19)
**Indigenous**	**1.71** (1.31, 2.24)	**1.49** (1.13, 1.98)	**1.46** (1.07, 1.97)	**1.36** (1.00, 1.85)	0.82 (0.47, 1.43)
**Married**	0.91 (0.73, 1.14)	0.94 (0.75, 1.18)	0.94 (0.74, 1.19)	0.95 (0.74, 1.20)	0.93 (0.73, 1.18)
**Urban**	**0.79** (0.63, 0.99)	0.85 (0.67, 1.07)	0.83 (0.64, 1.06)	0.86 (0.67, 1.10)	0.85 (0.66, 1.09)
**Education (** ***ref: No schooling*** **)**					
**Primary**		**0.70** (0.55, 0.90)	**0.72** (0.56, 0.94)	**0.76** (0.58, 0.99)	0.77 (0.59, 1.01)
**Secondary +**		**0.48** (0.33, 0.71)	**0.53** (0.35, 0.78)	**0.58** (0.39, 0.88)	**0.60** (0.40, 0.91)
**Work**		**0.54** (0.42, 0.70)	**0.66** (0.51, 0.87)	**0.65** (0.50, 0.86)	**0.64** (0.49, 0.85)
**Income sources (** ***ref: 0*** **)**					
**1–2**		0.80 (0.63, 1.02)	**0.77** (0.60, 0.99)	0.78 (0.61, 1.01)	0.78 (0.60, 1.00)
**3+**		**0.59** (0.43, 0.81)	**0.52** (0.37, 0.73)	**0.54** (0.38, 0.77)	**0.54** (0.38, 0.77)
**Variables**	**Model 1**	**Model 2**	**Model 3**	**Model 4**	**Model 5**
**Physical Limits (** ***ref: 0*** **)**					
**1–2**			**1.44** (1.05, 1.97)	**1.40** (1.01, 1.92)	**1.42** (1.03, 1.96)
**3+**			**2.46** (1.70, 3.57)	**2.29** (1.57, 3.34)	**2.27** (1.55, 3.30)
**HBP**			**1.40** (1.11, 1.76)	**1.41** (1.12, 1.78)	1.15 (0.84, 1.58)
**Diabetes**			**1.68** (1.27, 2.23)	**1.73** (1.31, 2.29)	**1.76** (1.32, 2.33)
**CHD**			**2.90** (2.12, 3.96)	**2.98** (2.17, 4.10)	**2.90** (2.10, 4.00)
**Arthritis**			**2.19** (1.75, 2.73)	**2.18** (1.74, 2.72)	**1.65** (1.22, 2.24)
**Asthma**			**1.99** (1.51, 2.62)	**1.92** (1.45, 2.54)	**1.94** (1.47, 2.58)
**Exercise (** ***ref: 0 weekly*** **)**					
**1–3 weekly**				0.76 (0.56, 1.04)	0.77 (0.57, 1.05)
**4+ weekly**				**0.60** (0.44, 0.83)	**0.60** (0.43, 0.83)
**Smoke (** ***ref: Non-smoker*** **)**					
**Previous smoker**				1.28 (0.91, 1.79)	1.25 (0.89, 1.76)
**Current smoker**				1.21 (0.87, 1.68)	1.20 (0.86, 1.67)
**Interaction effects**					
**HBP by Ethnic**					
**HBP by Chinese**					**2.09** (1.17, 3.76)
**HBP by Indian**					0.96 (0.43, 2.10)
**HBP by Indigenous**					1.45 (0.81, 2.59)
**Arthritis by Ethnic**					
**Arthritis by Chinese**					1.32 (0.74, 2.37)
**Arthritis by Indian**					**3.32** (1.52, 7.24)
**Arthritis by Indigenous**					1.74 (0.95, 3.22)

Note: Figures in brackets show 95 percent confidence intervals. Odds ratio in bold indicate significance at 0.05.

In the third model, the odds of poor self-rated health increased with the number of physical limitations. Respondents with 3 or more limitations were 2.5 times more likely to report poor health than those with no physical limitation. The odds of poor self-rated health were also higher in the presence of each of the 5 NCDs, with the odds ratios ranging from 1.4 to 2.9. Respondents suffering from CHD were almost 3 times more likely to report poor health than those without this disease. Controlling for other variables, the effects of gender, ethnicity and SES remained significant, but the effect of age had become insignificant. When lifestyle factors were included in the fourth model, only exercise was significant in predicting the odds of poor self-rated health. Respondents who exercised more than 3 times a week were 1.7 times less likely to report poor health, compared to those who did not exercise. The effects of gender, ethnicity, SES, physical limitations and the 5 diseases remained significant after taking into account lifestyle factors.

There were significant interactions between ethnicity and 2 diseases: HBP and arthritis. Therefore results on the effects of ethnicity were interpreted conditional upon these diseases. Chinese respondents who were suffering from HBP were more likely to report poor health, compared to Malays and other ethnic groups who were suffering from the similar disease. Among those suffering from arthritis, the Indians were more likely to report poor health than the rest. Controlling for these significant interactions, the effects of gender, SES, physical limitations and the 3 other diseases remained significant, with little changes in the odds ratios.

## Discussion

NCDs are the major causes of mortality globally. In Malaysia, the Indian males who had the highest prevalence of life threatening diabetes had the lowest life expectancy. In 2010, life expectancy at births among all males and females in Malaysia stood at 71.9 years and 77.0 years respectively while that for Indian males was only 68.0 years [32]. Hence, reducing NCDs will likely narrow the ethnic differentials in mortality and life expectancy.

Among the five NCDs examined in this study, arthritis was most common, followed by HBP, diabetes, asthma and CHD. The overall prevalence rate of arthritis and diabetes among older Malaysians are rather close to the level in the developed countries such as the United States of America [3], [33]. However, the overall prevalence of HBP among older Malaysians was still lower than that in USA which stood at around 60 to 70 percent for the period 1999–2008 [4], [34].

Older Malaysian Indians had very high rate of diabetes, and they were much more likely than those from other ethnic groups to have this disease. These findings corroborate with other reports of higher prevalence in diabetes among people of Indian ethnic origin. Genetic susceptibility to diabetes, and urbanization which results in a sedentary lifestyle are several risk factors alluded to in these studies [5], [35], [36]. Age is also a likely factor in the case of type 2 diabetes [3]. A recent breakthrough in scientific research has suggested a link between type 2 diabetes and aging [37]. The study found that age-associated reductions in mitochondrial function can cause muscle insulin resistance, which is a characteristic of the disease. Diabetes is also known to increase the risk of cardiovascular diseases [38]. Our findings show that Indians were also more likely than others to suffer from CHD.

Tobacco use is a probable cause of HBP and asthma. Indigenous groups and, to a lesser extent, the Malays tended to have smoking habits as compared to the Chinese and Indians. This may explain their higher likelihood of suffering from HBP and asthma. Lack of exercise may also contribute to the risk of HBP and arthritis among Indigenous groups, although there are other likely risks factors which need further investigation. Besides being predominantly rural, Indigenous groups are also disadvantaged in terms of socio-economic conditions. The Indigenous groups are lagging behind the others in educational attainment and they are concentrated in the rural areas where health services are not as good as in the urban areas. Older Indigenous people are likely to have worked as low-skilled workers in rural sectors, and these posed as risk factors for higher prevalence of arthritis.

CHD has the strongest adverse effect on poor self-rated health, followed by arthritis, asthma, diabetes and HBP. This study finds that the older Chinese tended to report better health, and they generally had lower prevalence of NCDs as compared to the other ethnic groups. Indians and Indigenous people were much more likely to report poor health, even after taking account SES, health and lifestyle variables. However, ethnic differentials in poor self-rated health were also found to be conditional upon certain diseases. Suffering from HBP was more debilitating to the Chinese, as compared to the other ethnic groups. On the other hand, older Indians were more likely than those from other ethnic groups to be adversely affected by arthritis.

Among those with HBP, cultural differentials may explain the higher propensity among Chinese to report poor health, as compared to other ethnic groups. Chinese, in general, have a preference for ‘tastier’ food containing high levels of sodium. Yet, changing dietary patterns among Chinese is not an easy task [39]. Having to change their diet in order to have better health, may instead have an adverse effect on their well-being.

A good knowledge of arthritis is needed for an explanation of the higher odds among Indians in reporting poor health, especially those who have this disease. Osteoarthritis and rheumatoid arthritis are among the common types of arthritis, though, the latter type is more painful and severe [40]. It has been found that prevalence in rheumatoid arthritis in India was higher than that in China and Indonesia [41]. Therefore, in our present study, a possible reason why Malaysian Indians were more affected by arthritis may be due to higher cases of rheumatoid arthritis, compared to the Chinese, Malays and Indigenous people.

There are pronounced gender differentials in the prevalence of NCDs. Our findings corroborate with reports that older females were more likely than older males to suffer from arthritis [42]. Older females, in general, were also more likely than older men to suffer from HBP [43], with the exception of the Chinese [44]. The higher odds of suffering from asthma among males may be due to higher tobacco use (Non-smokers: Females –86%, Males –32%). However, gender differentials in self rated health are not consistent with findings from most studies. Our findings suggest older females were less likely than older males to report being in poor health run contrary to findings from several countries [19], [20], [45], including previous studies in Malaysia [11]. However, it is to be noted that the non-significant observed gender differential in self rated health has become significant, after taking into account SES variables, which the earlier Malaysian study had not accounted for. Moreover, adjusting for chronic diseases may also alter gender differentials in self-rated health [46], [47].

Our findings show a strong inverse relation between SES variables and poor self-rated health, and these are consistent with findings from other studies [17], [18], [19]. Higher educational attainment, having multiple sources of income and continued participation were found to lead to positive self-rated health among older persons. Our finding on the effects of work on health status reaffirms the “healthy worker effect” (HWE) [48]. Social connections developed through employment may have a positive psychological effect on health. But in our case, it is also likely that healthier older persons were more likely to continue working [49]. As seen in our analyses, Malaysians who were working were less likely to suffer from the 5 diseases. Nevertheless, the significance of SES variables does imply the importance of finances in accessing healthcare services and treatment for illnesses.

Interestingly, having multiple sources of income is positively predictive of HBP and CHD. It is probable that more sources of income led to more affluent eating habits, and thus increased the risks of NCDs. A previous study on the prevalence of obesity in Malaysia which had included younger age groups found that Malays and Indians had higher prevalence of obesity, at around 13.5% each. The prevalence rate of obesity among Chinese and Indigenous groups in Sabah and Sarawak was relatively lower at 8.5%, 7.3% and 10.8% respectively. Gender differentials were also apparent as females were more likely to be obese at 13.8%, compared to males at 9.6% [50].

This study has several limitations. The analysis was confined to 5 NCDs as these were the only ones covered in the 2004 MPFS. A more comprehensive study of the health problems of older people would include the prevalence of obesity, other cardiovascular diseases, chronic respiratory diseases and cancer. Furthermore, self-reports of the 5 NCDs were not confirmed through medical personnel. Thus, the reliability of these self-reports may be questionable. However, studies conducted by the Malaysian Ministry of Health, which followed medical guidelines, reported almost similar statistics. For instance, according to the Third National Health and Morbidity survey in 2006, the prevalence of HBP and diabetes among the Malaysian adult population aged 30 and above, was 42.6% and 14.9% respectively [51]. Another study based on this survey had reported that 49.3% of older Malaysians aged 60 and above were aware of their HBP status, whereas 42.2% were receiving treatment for HBP [52]. Due to unavailable data, the number of sources of income was used to measure the relative financial position of the respondents, as the actual income was collected only for those who were currently working. The use of cross-sectional data precludes an analysis of the causal effects of lifestyle factors on the health status of older persons over a longer period. Given the dynamic changes in socio-economic conditions in the country during the last one decade [53], the 2004 MPFS is rather outdated. Nevertheless, this data set allows for an examination of health problems of older Malaysians of different ethnic origin. An update and more vigorous analysis will be carried out in 2015 with the release of the 2014 Malaysian round of survey.

“Prevention is better than cure” or so the old adage goes. Health intervention programs need to be stepped up to promote healthy diet and lifestyle among Malaysians from all age groups to contain the debilitating effects of NCDs [1]. Appropriate customized educational intervention according to ethnic groups and gender to help people understand and cope with NCDs will be more effective in containing these diseases [54]. Promoting active and productive aging could also lead to an improvement in health among the older people, while allowing them to be self-reliant in health care cost should the need arises.
